# Accelerated Diffusion-Weighted MRI of Rectal Cancer Using a Residual Convolutional Network

**DOI:** 10.3390/bioengineering10030359

**Published:** 2023-03-14

**Authors:** Mohaddese Mohammadi, Elena A. Kaye, Or Alus, Youngwook Kee, Jennifer S. Golia Pernicka, Maria El Homsi, Iva Petkovska, Ricardo Otazo

**Affiliations:** 1 Department of Medical Physics, Memorial Sloan Kettering Cancer Center, New York, NY 10065, USA; 2 Department of Radiology, Memorial Sloan Kettering Cancer Center, New York, NY 10065, USA

**Keywords:** diffusion-weighted MRI, rectal cancer, deep learning, denoising

## Abstract

This work presents a deep-learning-based denoising technique to accelerate the acquisition of high *b*-value diffusion-weighted MRI for rectal cancer. A denoising convolutional neural network (DCNN) with a combined L1–L2 loss function was developed to denoise high *b*-value diffusion-weighted MRI data acquired with fewer repetitions (NEX: number of excitations) using the low *b*-value image as an anatomical guide. DCNN was trained using 85 datasets acquired on patients with rectal cancer and tested on 20 different datasets with NEX = 1, 2, and 4, corresponding to acceleration factors of 16, 8, and 4, respectively. Image quality was assessed qualitatively by expert body radiologists. Reader 1 scored similar overall image quality between denoised images with NEX = 1 and NEX = 2, which were slightly lower than the reference. Reader 2 scored similar quality between NEX = 1 and the reference, while better quality for NEX = 2. Denoised images with fourfold acceleration (NEX = 4) received even higher scores than the reference, which is due in part to the effect of gas-related motion in the rectum, which affects longer acquisitions. The proposed deep learning denoising technique can enable eightfold acceleration with similar image quality (average image quality = 2.8 ± 0.5) and fourfold acceleration with higher image quality (3.0 ± 0.6) than the clinical standard (2.5 ± 0.8) for improved diagnosis of rectal cancer.

## 1. Introduction

According to the American Cancer Society, an estimated 44,850 individuals will be newly diagnosed with rectal cancer in 2022 in the US, out of 150,030 newly diagnosed colorectal cancer [1]. Treatment for locally advanced rectal cancer is a total neoadjuvant treatment, and about 30% of patients will respond with a complete pathologic response [2]. MRI is the preferred imaging modality to diagnose rectal cancer [3,4,5,6], and T2-weighted imaging and diffusion-weighted imaging (DWI) are the most frequently used methods [7], presenting high accuracy in the detection of cases with complete clinical response. It has been shown that DWI increased the sensitivity of detecting pathological clinical response (+27%) [8]. However, the specificity of DWI alone and combined with T2WI remained low at 63% and 56%, respectively [9]. Hence, there is a need to improve the accuracy of DWI for rectal cancer by improving image quality.

DWI for rectal cancer is typically performed via single-shot echo planar imaging (ss-EPI) acquisition [10]. ss-EPI is the fastest technique to acquire DWI data and presents very low sensitivity to macroscopic organ motion [11,12]. However, ss-EPI has limitations in terms of spatial resolution and SNR and presents geometric distortions. First, since all k-space lines need to be acquired within the duration of one shot, spatial resolution is compromised, and the relatively long duration of the signal readout results in geometric distortions and signal pile-ups due to sensitivity to magnetic field inhomogeneities [13]. Second, the application of diffusion-weighted magnetic field gradients reduces signal-to-noise ratio (SNR) proportionally to the diffusion weight or *b*-value, and significant SNR degradation is expected for high *b*-value images [14]. To compensate for the inherent low SNR, the acquisition is repeated multiple times (usually 16 times in the rectum), and images are averaged to reduce noise [15]. This is a practical but very expensive solution, as it increases scan time proportionately to the number of repetitions.

Several techniques have been proposed to accelerate DWI and thus compensate for the additional scan time required for repeated acquisitions. The first group of techniques aims to undersample k-space and use advanced reconstruction techniques such as parallel imaging, compressed sensing, or deep learning to reconstruct unaliased images from undersampled k-space data. Parallel imaging techniques, such as Sensitivity Encoding (SENSE) [16] and generalized autocalibrating parallel acquisitions (GRAPPA) [17,18], have been extensively applied to reduce the readout duration and thus reduce distortions or increase spatial resolution in DWI [19,20,21,22,23]. Simultaneous multislice (SMS) techniques enable through-plane undersampling by simultaneously acquiring multiple slices. The combination of through-plane SMS acceleration and in-plane parallel imaging acceleration has led to further improvements in spatial resolution and scan time reductions [24]. Compressed sensing has also been applied to accelerate diffusion MRI, but mainly diffusion tensor imaging (DTI), which has higher dimensionality and thus is a better candidate than ss-EPI [25,26].

Another obstacle to the application of compressed sensing is the need for random undersampling, which is challenging for an EPI readout. The second group of techniques reduces the number of repetitions and denoises the resulting images to compensate for the reduced averaging. Several denoising techniques have been applied to DWI data to reduce the number of repetitions, including joint reconstruction and denoising of complex-values images [27], data-driven principal component analysis (PCA) [28], and model-based Bayesian denoising of magnitude images [29].

Deep learning techniques have been recently proposed for both the reconstruction and denoising of DWI data. The goal of deep learning is to train a convolutional neural network (CNN) to reconstruct undersampled k-space DWI data or to denoise diffusion images acquired with fewer repetitions. Higher undersampling factors (up to a factor of 5) were reported using deep learning reconstruction of ss-EPI data [30]. Deep learning has also been applied to reduce the number of repetitions for high *b*-value DWI in the brain [31] and prostate [32] by training a CNN that maps a noisy image acquired with a few repetitions to a denoised image that resembles the one acquired with a large number of repetitions.

This work proposes to develop a denoising convolutional neural network (DCNN) trained with rectal cancer patient data to accelerate the acquisition of high *b*-value DWI data in the rectum. The network will be trained using rectal cancer patient data to include tumor variability and rectum-specific physiological variables such as gas in the rectum and peristalsis, which make the problem of DWI in the rectum more challenging than in the brain or prostate. A modified loss function is proposed for training, where a linear combination of L1 and L2 terms is employed to obtain an appropriate compromise between denoising and smoothing [33,34]. Results are evaluated quantitatively using the peak signal-to-noise ratio (PSNR) and structural similarity (SSIM) and qualitatively by expert body radiologists.

## 2. Materials and Methods

### 2.1. Data Acquisition

Raw k-space data from 120 patients were collected retrospectively on 13 3T MRI scanners (GE Healthcare, Waukesha, WI, USA) with approval from the institutional review board. Eighty-five datasets (3079 images for different slices in the acquisition, 70%) were used for network training, 15 datasets (608 images, 12.7%) were used for cross-validation, and 20 datasets (760 images, 16.9%) were used for testing different versions of the deep learning approach. DWI data were acquired using a ss-EPI DWI pulse sequence as part of our standard rectal MRI examination. Diffusion gradients were applied simultaneously along the three spatial dimensions x, y and z with *b* = 0, 50 (low *b*-value) and 800 (high *b*-value) s/mm2. Low *b*-value data acquisition was performed with 2 or 4 repetitions (NEX = 2 or 4) and high *b*-value with 16 repetitions (NEX = 16). Relevant imaging parameters include the following: field of view (FOV) = 16–20 cm, phase-encoding FOV coverage = 100%, slice thickness = 5 mm, space between slices = 1 mm, number of slices = 30–50, TR = 6–8 s, TE = 54–74 ms (shortest TE available using partial Fourier as determined by the vendor implementation of the sequence), in-plane matrix size = 140 × 140.

### 2.2. Image Reconstruction

**Figure 1:**
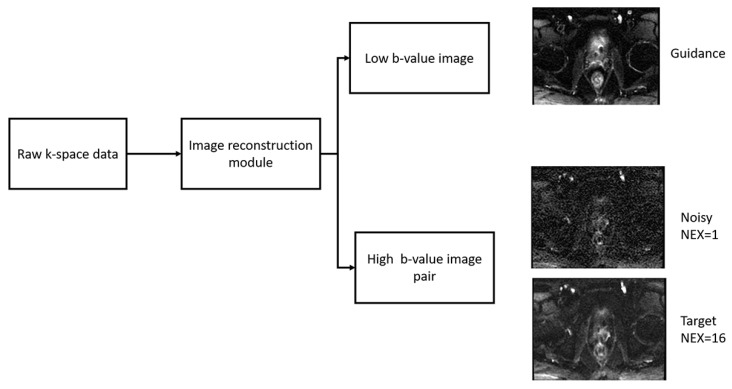
Offline reconstruction of DWI raw data. Raw k-space data from every patient was reconstructed to produce three images: guidance (low *b*-value DWI), noisy (high *b*-value DWI reconstructed with NEX = 1, 2, and 4), and reference (high *b*-value DWI reconstructed with NEX = 16).

Images were reconstructed offline in MATLAB (Mathworks, Natwick, MA, USA) using the EPI reconstruction functions in the GE Healthcare Orchestra Reconstruction Software Development Toolkit. Images for each repetition (NEX) were reconstructed separately and averaged to produce three types of images (Figure 1): guidance (low *b*-value DWI with NEX = 2 or 4), reference (high *b*-value DWI reconstructed with NEX = 16) and noisy (high *b*-value DWI reconstructed with NEX = 1, 2, and 4 to test acceleration factors of 16, 8, and 4, respectively). Noisy and reference images were normalized to the maximum value of signal intensity among all slices of the reference images, and low *b*-value images were normalized to the maximum intensity of all slices. ADC maps were calculated pixel-wise by using the following equation:

(1) $$ADC=-\frac{1}{∆b}\ln\left(\frac{s_{b}}{s_{0}}\right),$$

where $s_{0}$ is the signal at low *b*-value, $s_{b}$ is the signal at a high *b*-value, and ∆b is the difference between high and low *b*-value.

### 2.3. Denoising Convolutional Neural Network (DCNN)

**Figure 2:**
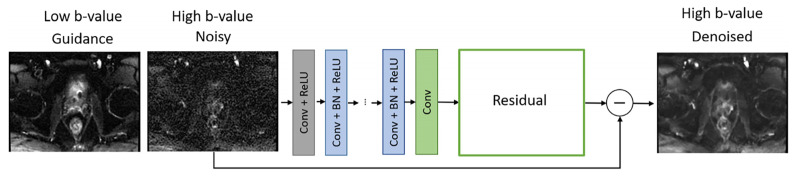
Denoising convolutional neural network (DCNN) architecture. The DCNN denoises the high *b*-value image using the low *b*-value image as a guide. The DCNN is based on residual learning and uses a loss function that combines an L2-term and an L1-term for an appropriate tradeoff between denoising and smoothing.

DCNN has two inputs that were given by the high *b*-value image to be denoised and the low *b*-value image that serves as an anatomical guide, and the output is the denoised high *b*-value image (Figure 2). DCNN uses residual learning to separate noise from a noisy image, which was demonstrated to improve robustness of the denoising process by including the feedback loop [35]. DCNN is composed of 64 layers. The first layer generated 64 feature maps using 3 × 3 × 2 convolution filters and nonlinearity rectified linear units (ReLU). In layers 2 to 63, 64 filters of size 3 × 3 × 64 were used. Batch normalization was implemented between convolution and ReLU. The output was reconstructed in the last layer using one filter of size 3 × 3 × 64. Input images were converted to 60 × 60 patches. Pair of patches were rotated and flipped during mini-batch learning.

DCNN is trained by minimizing a loss function given by a linear combination of an L2 term (sum of all squared differences between denoised and reference images) and an L1 term (sum of absolute differences between denoised and reference images):

(2) $$L_{L1-L2}=\sum_{n=1}^{N_{training}}\frac{1}{2}{\left\|d_{n}-r_{n}\right\|}_{2}^{2}+\lambda \sum_{n=1}^{N_{training}}{\left\|d_{n}-r_{n}\right\|}_{1},$$

where *d_n_* is the output of the network, *r_n_* is the reference high *b*-value image, *d N_training_* is the number of training datasets, and *λ* is the weighting factor for the L1 term (right-hand-side) relative to the L1 term (left-hand-side). DCNN was trained for 18 epochs. The loss was calculated on the validation set every third epoch, and it decayed consistently. To select the value of l, five different networks with weighting factors = 2, 4, 5, 6, and 10 were trained. Denoised images from one test case using the five different networks were evaluated by the expert body radiologist in terms of denoising and smoothing. The selected DCNN was then applied to the testing cases using noisy images with NEX = 1, 2, and 4, which corresponds to acceleration factors of 16, 8, and 4, respectively. The denoised output of these three networks was then evaluated by quantitative metrics and qualitatively by expert body radiologists.

### 2.4. Quantitative Evaluation

Peak signal-to-noise ratio (PSNR) [36] and structural similarity (SSIM) [36] of noisy and denoised images acquired with NEX = 1, 2, and 4 with respect to reference images acquired with NEX = 16 were computed to assess image quality quantitatively. PSNR was computed as follows [36]:

(3) $$PSNR(d,r)=10log\frac{d_{max}}{MSE(d,r)}=10log\frac{d_{max}}{\frac{1}{n}\sum_{i}{\left(d_{i}-r_{i}\right)}^{2}},$$

where *d* is the noisy/denoised image, *r* is the reference, *i* is an index for the pixels in *d*, and *r, n* is the number of pixels, *d_max_* is the peak intensity in *d*, and MSE is the mean squared error. SSIM was computed as follows [36]:

(4) $$SSIM\left(d,r\right)=I(d,r)C(d,r)S(d,r),$$

where $I\left(d,r\right)=\frac{2\mu_{d}\mu_{r}}{u_{d}^{2}+u_{r}^{2}}$ is the luminance comparison function, $C\left(d,r\right)=\frac{2\sigma_{d}\sigma_{r}}{\sigma_{d}^{2}+\sigma_{r}^{2}}$ is the contrast comparison function, $S\left(d,r\right)=\frac{\sigma_{dr}}{\sigma_{d}\sigma_{r}}$ is the structure comparison function, *μ* is the mean value, *σ* is the standard deviation, and *σ_dr_* is the covariance between *d* and *r*.

PSNR and SSIM were computed for different loss functions, including L2-only, L1-only, and joint-L1–L2. To assess the local image quality in the rectal region, PSNR and SSIM were computed in a region of interest surrounding the rectum.

### 2.5. Qualitative Evaluation by Expert Body Radiologist

Qualitative evaluation was performed by two diagnostic body radiologists with 11 and 7 years of experience. Radiologists were blinded to the project goals and independently reviewed noisy (NEX = 1,2,4), reference (NEX = 16), and denoised (NEX = 1,2,4) high *b*-value images presented in a randomized order. Scenarios where one type of image would be immediately followed by another type of image for the same patient were avoided by manually adjusting the order. A four-point Likert scale (Table 1) was employed in a similar way to previous studies [37,38,39]. The scale is 1 (Nondiagnostic/poor) to 4 (Excellent), and the readers scored images based on overall image quality, rectum margin and rectal wall layers demarcation, noise suppression, and image sharpness.

## 3. Results

**Figure 3:**
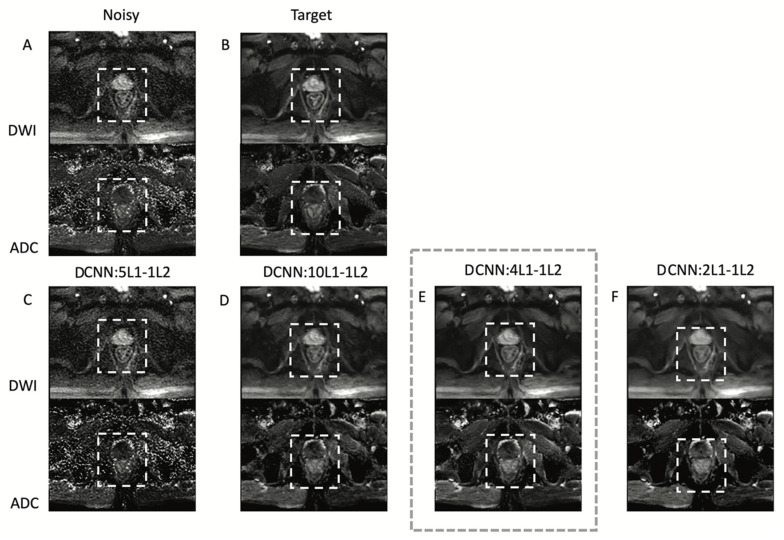
DCNN with different weighting factors for the L1–L2 combination loss function. (**A**) Noisy high *b*-value image reconstructed with NEX = 1 corresponding to an acquisition time of 22 s. (**B**) Target high *b*-value image with NEX = 16 corresponding to 370 s acquisition time. (**C**–**F**) denoised images acquired with NEX = 1 using 2, 4, 5, and 10 L1 weighting factors. The square with dashed lines shows the region of interest used to compute quantitative image quality metrics. A body radiologist selected the network with a weighting factor of 4 significantly as the one with the best balance between denoising and delineation of the rectal wall.

Figure 3 shows the performance of DCNN for different weighting factors of the L1 term with respect to the L2 term in the loss function. The network with a weighting factor of 4 was selected by an expert body radiologist as the one with the best balance between denoising and delineation of the rectal wall. Using lower weights than 4 on L1 with respect to L2 (e.g., 2) result in oversmoothed images, while using higher weights than 4 on L1 with respect to L2 (e.g., 5 and 10) result in noisier images. The L1–L2 network with a weighting factor of 4 was used from this point forward.

**Figure 4:**
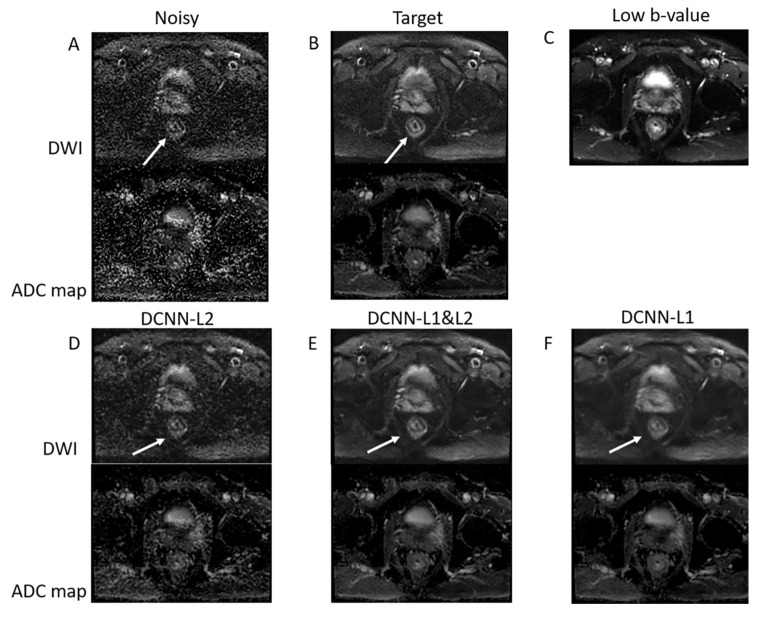
DCNN with different loss functions. (**A**) Noisy high *b*-value image reconstructed with NEX = 1 corresponding to an acquisition time of 22 s. (**B**) Target high *b*-value image with NEX = 16 corresponding to 370 s acquisition time. (**C**) Low *b*-value image used as a guide. (**D**–**F**) Denoised images were acquired with NEX = 1 using L2, joint L1–L2, and L1 loss functions. The joint L1–L2 loss function presents a better compromise between denoising and smoothing than L2 and L1 alone. Specifically, rectal wall layers (shown by the white arrow) are clearly improved when using the joint L1–L2 loss function.

The performance of different loss functions (L1 alone, L2 alone, and joint L1–L2) to denoise high *b*-value DWI data acquired with only one repetition in a representative patient with rectal cancer is presented in Figure 4. The joint L1–L2 loss function outperforms the L2 and L1 loss functions. Specifically, the L2 loss function presents residual noise, and the L1 loss function presents blurring. The use of a combined L1 and L2 loss function can improve the tradeoff between denoising and smoothing, presenting a denoised image with preserved anatomical details. This result represents a 16-fold acceleration in the acquisition of high *b*-value DWI data, reducing the acquisition time from 370 s to 22 s.

Quantitative performance between different loss functions is presented in Table 2 using PSNR and SSIM averaged over all slices for all patients in the testing group. Deep learning denoising presented higher PSNR and SSIM than the noisy images for all loss functions, as expected. In addition, denoised images with L1–L2 loss function presented the overall highest PSNR and SSIM, which agrees with the analysis performed by the body radiologist.

**Figure 5:**
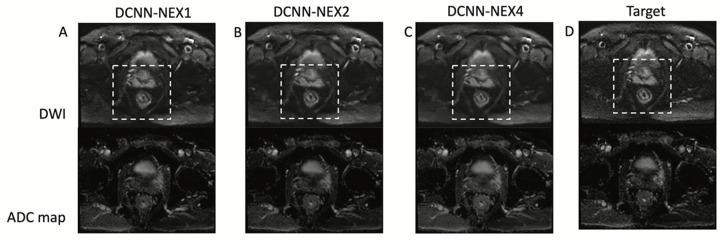
DCNN for different acceleration factors or a number of repetitions (NEX). DCNN results for NEX = 1 or 16-fold acceleration (**A**), NEX = two or eightfold acceleration (**B**) and NEX = four or fourfold acceleration (**C**). The dashed square shows the rectal area. The reference high *b*-value image (**D**) is shown for comparison purposes. DCNN presents high-performance denoising for all cases with a slight improvement for the case of NEX = 4.

Figure 5 shows the performance of DCNN for different numbers of repetitions (NEX) or acceleration factors. Denoising performance is high in all cases, with slightly better quality in NEX = 2 and NEX = 4 over NEX = 1, which was expected due to the use of more repetitions.

**Figure 6:**
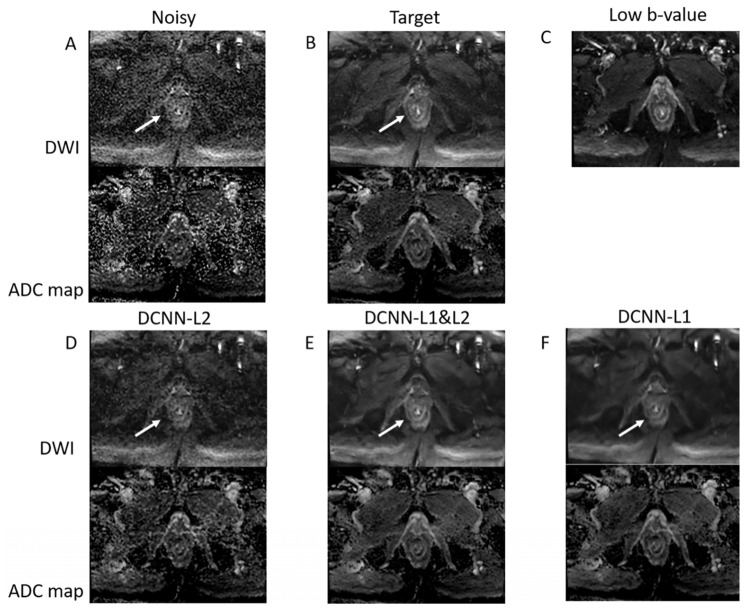
DCNN with different loss functions in another patient. (**A**) Noisy high *b*-value image reconstructed with NEX = 1 corresponding to an acquisition time of 22 s. (**B**) Target high *b*-value image with NEX = 16 corresponding to 370 s acquisition time. (**C**) Low *b*-value image used as a guide. (**D**), (**E**,**F**) denoised images acquired with NEX = 1 using L2, joint L1–L2, and L1 loss functions. In this case, the joint L1–L2 loss function also outperforms L1 and L2.

Figure 6 shows the performance of DCNN in a different patient. As in the previous case presented in Figure 3, the combined L1–L2 loss function outperforms both the L1 and L2 loss functions in terms of denoising and preservation of anatomical features.

**Figure 7:**
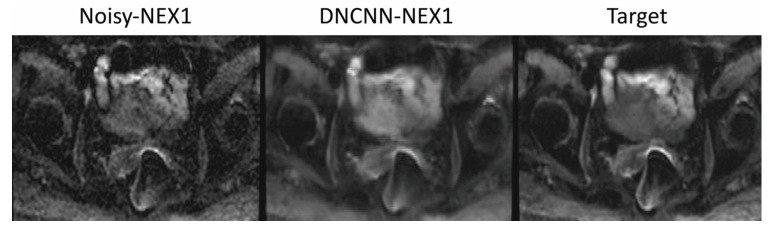
Patient with gas in the rectum that presents strong distortion artifacts. The DCNN-NEX1 (16-fold acceleration) presents improved image quality to the conventional reconstruction without denoising and similar image quality to the target with NEX = 16, despite the high acceleration.

Figure 7 shows the performance of DCNN in the presence of severe distortions produced by gas in the rectum. Despite the high acceleration, DCNN preserves the image quality of the target but improves the image quality of the conventional reconstruction without denoising. This example demonstrates the robustness of DCNN to severe distortions produced by the presence of gas in the rectum.

Quantification of ADC also resulted in high concordance between deep learning denoising and the reference. The mean ADC value in a region of interest around the rectum was 1.34 for DCNN-NEX1, 1.28 for DCNN-NEX2, 1.33 for DCNN-NEX4 and 1.29 for the reference NEX16 (ADC values are in $10^{-3}{mm}^{2}/s$).

Table 3 shows the results of the reader study to denoise high *b*-value images acquired with NEX = 1, 2, and 4 (acceleration factors of 16, 8, and 4, respectively). Overall, the denoised images received higher scores than the noisy images. Among different acceleration factors, images with 4-fold acceleration (NEX = 4) received the highest scores. Denoised images with 4-fold acceleration even received higher scores than the reference images. For example, in the overall image quality category, denoised images with NEX = 4 received a median score + interquartile range (IQR) of 3 ± 0.6 from reader 1, which means 50% of the scores spread from 2.4 to 3.6 in a 1–4 scale, and 3 ± 0.5 from reader 2, reference images (NEX = 16) received scores of 3 ± 1 from reader 1 and 2 ± 0.5 from reader 2. Reader 1 scored similar overall image quality (2.5 ± 0.5 on a 1–4 scale) between 16-fold (NEX = 1) and 8-fold (NEX = 2) accelerations, which were slightly lower than the reference (3.0 ± 1.0). Reader 2 scored similar quality between NEX = 1 and the reference (2.0 ± 0.5 for both), while better quality for NEX = 2 (3.0 ± 0.5).

## 4. Discussion

The proposed deep learning denoising of DWI in the rectum has strong clinical significance since MRI is the preferred imaging modality for rectal cancer imaging [40], and DWI is the most frequent imaging sequence to diagnose tumor response [3,4,5,6]. The clinical application goes beyond reducing total scan time and is expected to reduce sensitivity to the presence of gas in the rectum and to peristaltic motion, which are considered two of the major challenges for DWI in the rectum. This work is, to the best of our knowledge, the first to train a neural network using patient data with rectal cancer with the goal of reducing the number of repetitions and thus accelerating the acquisition and improving image quality.

Deep-learning-based denoising approaches have been shown to be promising in DWI applications in the brain [31] and prostate [32]. Clinical application to rectal cancer is more challenging than previous work in the brain and prostate due to the presence of gas-related motion in the rectum, which can result in severe image distortions, as shown in Figure 7. These distortions are different from blurring or ghosting artifacts from respiratory motion and present geometric deformations that can severely affect diagnosis. Longer acquisitions, such as conventional DWI performed with 16 repetitions, are more sensitive to gas in the rectum since the geometric distortions can be different in each repetition, and averaging will combine artifacts from different repetitions. An alternative to reducing the number of repetitions to 1 or 2 would be to co-register different repetitions before averaging at the expense of increased computational burden and the risk of registration-related blurring. The combination of shorter acquisitions with a reduced number of repetitions and deep learning denoising can enable the acquisition of diffusion images with reduced distortions and sufficient SNR for the robust use of DWI in patients with rectal cancer.

The utilization of a joint L1–L2 loss function achieved an improved tradeoff between denoising and smoothing than using L1 or L2 alone and resulted in better preservation of high-resolution features such as layers in the rectal wall. This type of loss function was already introduced in previous work for different types of applications [33,34]. This study confirms that a joint L1–L2 loss function presents higher performance than L1-alone or L2-alone.

Denoising of diffusion-weighted MRI is an active area of research. Previous to the application of deep learning, data-driven principal component analysis (PCA) and model-based Bayesian methods [41] were employed. PCA exploits correlations between repetitions to separate signal from noise. However, denoising capabilities are limited due to the different realization of noise in each repetition [28,42]. Bayesian methods added the Rician noise model for magnitude images and were demonstrated to outperform PCA. However, they are prone to smooth anatomical details in the denoised image and require many expensive iterations, which limits clinical implementation [27]. The application of deep learning represents a step forward in terms of denoising since the actual model between signal and noise can be learned directly from multiple datasets without the need for complicated mathematical models. Moreover, while training can be computationally expensive, once the CNN is trained, the application of the trained CNN is very fast and can even be performed in real-time [41].

The high acceleration obtained by the proposed deep learning method can be applied to more advanced DWI techniques, such as multishot EPI acquisition methods [43], which conventionally require longer scan times due to the acquisition of multiple shots. Reducing the number of repetitions can compensate for the extra time to acquire multiple shots, and therefore the combined result would be DWI with higher resolution and less distortion provided by multishot EPI and similar or shorter scan time provided by deep learning denoising. The proposed method can also be combined with deep learning reconstruction of undersampled k-space data [42] to denoise results after reconstruction.

This work also has limitations. The proposed method was validated in a small population of 20 patients with rectal cancer. Patient data were acquired at a single institution using scanners from a single manufacturer. Future work will explore multicenter studies using data acquired on scanners from different manufacturers. ADC comparison was performed using the mean value in a region of interest around the rectum. Future work will explore the segmentation of the rectal area to compute the ADC in different segments. Moreover, this study only evaluated image quality since the main goal was to demonstrate the feasibility of denoising high *b*-value DWI data. Future work will evaluate the clinical impact of the proposed technique, including a larger clinical population and assessment of DWI specificity for rectal cancer response after total neoadjuvant therapy.

## 5. Conclusions

This work demonstrates the application of deep learning denoising to reduce the number of repetitions in diffusion-weighted MRI of the rectum with a loss function that optimizes the tradeoff between denoising and smoothing. The proposed method enables eightfold acceleration with similar image quality (average image quality = 2.8 ± 0.5) and fourfold acceleration with improved image quality (3.0 ± 0.6) with respect to a reference acquired with 16 repetitions (2.5 ± 0.8), which can improve the diagnosis of rectal cancer.

## Figures and Tables

**Table 1:** Qualitative image evaluation criteria used by expert body radiologists.

Score	Overall Image Quality	Rectum Margin and Rectal Wall Layers Demarcation	Noise Suppression	Image Sharpness
1	Nondiagnostic/poor	No visualization or inability to trace structures clearly	Significant noise that hampers diagnostic capability of readers	Nondiagnostic, blurred, hampering diagnostic capability
2	Fair	Fair demarcation	Substantial noise with significant image quality degradation	Substantially blurred, not hampering diagnostic capability but low image quality
3	Good	Nearly complete and clear demarcation	Moderate noise	Mild blur with mild image quality degradation
4	Excellent	Complete and clear demarcation	Minimal noise without image quality degradation	Minimal or no blur

**Table 2:** PSNR and SSIM for high *b*-value noisy and denoised images (NEX = 1) with respect to the high *b*-value reference (NEX = 16) for different loss functions computed using all the patients in the testing set. Joint L1–L2 loss function presents the highest PSNR and SSIM, in agreement with the analysis performed by the body radiologist.

Loss Function	PSNR Denoised	PSNR Noisy	SSIM Denoised	SSIM Noisy
L1	84.13 ± 4.6	80.29 ± 5.1	0.89 ± 0.03	0.85 ± 0.1
L2	82.63 ± 5.2		0.90 ± 0.07	
Joint L1–L2	84.33 ± 5.1		0.94 ± 0.03	

**Table 3:** Qualitative image qualitative evaluation was performed by two expert radiologists. Median ± IQR scores are shown for each reader and image quality category.

	Image Quality	Rectum Margin and Rectal Wall Layers Demarcation	Noise Suppression	Image Sharpness
Reader 1				
Noisy, NEX = 1	2 ± 1	2 ± 1	2 ± 1	2.5 ± 0.5
Noisy, NEX = 2	2 ± 0.5	2 ± 0.5	2 ± 1	3 ± 0.5
Noisy, NEX = 4	2 ± 0.5	2 ± 0.5	2 ± 0.5	2 ± 0.5
Denoised NEX = 1	2.5 ± 0.6	2 ± 0.6	3 ± 1	2.5 ± 0.5
Denoised NEX = 2	2.5 ± 0.6	2 ± 0.1	3.5 ± 1	3 ± 0.5
Denoised NEX = 4	3 ± 0.6	3 ± 0.5	4 ± 0.5	3 ± 1
Target	3 ± 1	3 ± 1	3 ± 0.8	3 ± 1
Reader 2				
Noisy, NEX = 1	2 ± 0.6	2 ± 0.7	2 ± 1	2 ± 0.5
Noisy, NEX = 2	2 ± 0.5	2 ± 0.5	2.5 ± 0.5	2 ± 0.5
Noisy, NEX = 4	2 ± 0.5	2 ± 0.5	2.5 ± 0.5	2 ± 0.5
Denoised NEX = 1	2 ± 0.5	2 ± 0.5	3 ± 1	3 ± 0.5
Denoised NEX = 2	3 ± 0.5	3 ± 0.5	4 ± 0.5	3 ± 0
Denoised NEX = 4	3 ± 0.5	3 ± 0.5	4 ± 0.5	3 ± 0.5
Target	2 ± 0.5	2 ± 0.5	3 ± 0.5	2.5 ± 0.5

## Data Availability

We are planning to share the data, but it requires institutional authorization, which is currently in process.
